# Forsythoside A Modulates Zymosan-Induced Peritonitis in Mice

**DOI:** 10.3390/molecules23030593

**Published:** 2018-03-06

**Authors:** Xiao-Tian Zhang, Yue Ding, Ping Kang, Xin-Yu Zhang, Tong Zhang

**Affiliations:** 1 School of Pharmacy, Shanghai University of Traditional Chinese Medicine, Shanghai 201203, China; zhangxt92@163.com (X.-T.Z.); dingyue-2001@hotmail.com (Y.D.); 2 Experiment Center of Teaching & Learning, Shanghai University of Traditional Chinese Medicine, Shanghai 201203, China; 3 Headmaster’s office, Shanghai University of Traditional Chinese Medicine, Shanghai 201203, China; konnie1984@hotmail.com

**Keywords:** forsythoside A, zymosan, peritonitis, inflammation

## Abstract

Acute inflammation is a protective response of the host to physical injury and invading infection. Timely treatment of acute inflammatory reactions is essential to prevent damage to organisms that can eventually lead to chronic inflammation. Forsythoside A (FTA), an active constituent of *Forsythia suspensa*, has been reported to have anti-inflammatory, antioxidant, and antibacterial properties. Despite increasing knowledge of its anti-inflammatory effects, the mechanism and the effects on acute inflammation are poorly understood. This study is aimed at exploring the pro-resolving effects of FTA on zymosan-induced acute peritonitis. FTA significantly alleviated peritonitis as evidenced by the decreased number of neutrophils and levels of tumor necrosis factor alpha (TNF-α), interleukin-6 (IL-6), and monocyte chemoattractant protein-1 (MCP-1) in the peritoneal cavity, without interfering with interleukin-10 (IL-10). FTA showed marked regulation of inflammatory cytokines and chemokine levels in zymosan-stimulated RAW 264.7 macrophages. Moreover, FTA could suppress the activation of NF-κB. In conclusion, FTA alleviated zymosan-induced acute peritonitis through inhibition of NF-κB activation.

## 1. Introduction

Inflammation can usually be divided into two major categories according to the course of disease: acute inflammation and chronic inflammation. Acute inflammation of abrupt onset, short duration, with exudative lesions is characterized by granulocyte infiltration of inflammatory cells. More concretely, burns, wounds, infections, and other conditions would lead to acute inflammation, resulting in the release of a large number of inflammatory factors, which would cause systemic inflammatory response syndrome (SIRS), which could further develop into multiple organ dysfunction syndrome (MODS), eventually causing death [1,2,3].

Acute inflammation in healthy individuals is self-limited and a dynamic process. According to its characteristics, it can be divided into several different periods. Firstly, in response to injury or infection, polymorphonuclear neutrophil (PMN) and eosinophil migrate to inflammatory sites to neutralize and eliminate potentially injurious stimuli. Accompanied by the rapid proliferation of PMN and inflammatory cell infiltration, next is the peak of inflammation in the sequence of events, which makes the exit routes available to PMN, including cell clearance and monocyte-derived macrophages. Then the apoptotic PMN are cleared by macrophages through phagocytosis. Meanwhile, TGF-β1 is released, which has anti-inflammatory and repair effects on the site of inflammation. Finally, the macrophages complete the task and depart through lymphatic drainage. If all these process occur, then acute inflammation will resolve [4,5,6].

**Figure 1:**
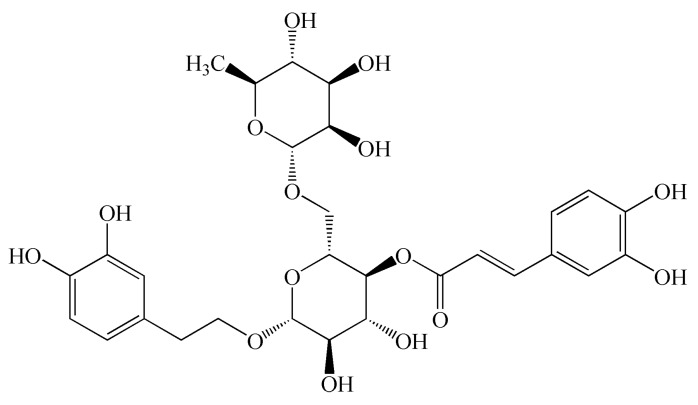
Chemical structural formula of forsythoside A.

As a commonly-used traditional Chinese medicine, *Forsythia suspensa* has a wide range of pharmacological activities [7,8]. It is worth noting that it has very strong antibacterial and anti-inflammatory effects [9]. Previous study showed that FTA (Figure 1) extracted from *Forsythia suspensa* provided a very significant anti-inflammatory activity [10]. It has also been suggested that FTA has neuroprotective, antitumor, antiviral, and antioxidation effects [11,12,13]. Moreover, it was reported that FTA presented antipyretic and vasodilatation effects [14]. For its anti-inflammatory mechanisms, it has been described that FTA could inhibit NF-κB-mediated inflammation response, decrease IL-1β, NO, TNF-α, and IL-10 levels, enhance IL-2 secretion, and up-regulate the expression of Nrf2 and heme oxygenase-1 [15,16,17,18,19,20]. During this research, we further explored the anti-inflammatory action of FTA, especially its therapeutic effect on acute peritonitis, and explored the underlying mechanisms.

## 2. Results

### 2.1. Treatment with FTA Decreases Neutrophils Numbers in the Peritoneal Cavity after Induction of Peritonitis

**Figure 2:**
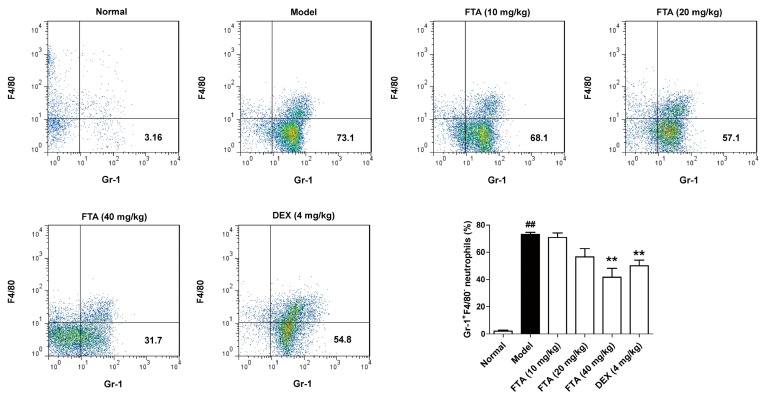
Influence of FTA on the inflammatory response in zymosan-induced peritonitis. Four hours after the injection of the zymosan, cells were extract from the peritoneal lavage fluid. The proportion of neutrophils (Gr-1^+^F4/80^−^) was measured by flow cytometry. Data were expressed as means ± SEM (*n* = 8). ** *p* < 0.01 vs. Model; ^##^ *p* < 0.01 vs. Normal.

FTA has been used as a traditional anti-inflammatory agent, however, the time course over which FTA resolves inflammation has not been reported. To evaluate the inflammation-resolution of FTA, an acute peritonitis model was established in male C57BL/6J mice. There was a significant increase in neutrophils (Gr-1^+^F4/80^−^) at the site 4 h after zymosan i.p. injection. Compared with the model group, FTA (40 mg/kg) and DEX treatment reduced neutrophil cell numbers (Figure 2).

### 2.2. FTA Treatment Influences Cytokines Levels in the Peritoneal Cavity

**Figure 3:**
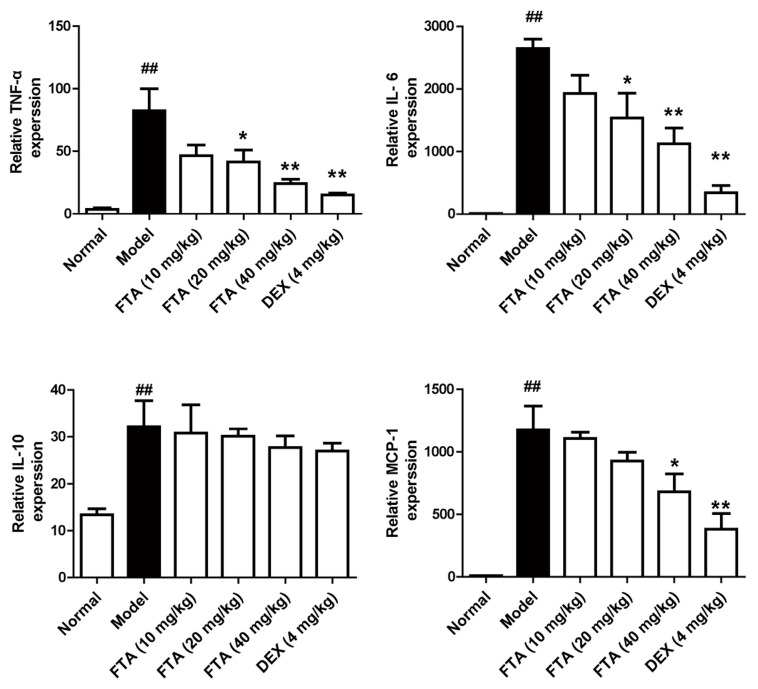
FTA treatment influences cytokines levels in peritoneal cavity. Levels of cytokines (TNF-α, IL-6, IL-10 and MCP-1) were determined by ELISA. Data were expressed as means ± SEM, *n* = 8. * *p* < 0.05, ** *p* < 0.01 vs. Model; ^##^ *p* < 0.01 vs. Normal.

In acute peritonitis, inflammatory cytokines and chemokines are closely implicated with the peritonitis pathogenesis. We further observed the levels of cytokines in peritoneal cavity of zymosan-induced peritonitis in mice by ELISA. The results suggested that animals treated with FTA (40 mg/kg) displayed lower levels of inflammatory cytokines (TNF-α and IL-6) and chemokine (MCP-1) than the model group, whereas it had little effect on the IL-10 level. DEX had similar effects (Figure 3).

### 2.3. FTA Reduces the Expressions of Cytokines in Zymosan-Stimulated RAW 264.7 Cells

**Figure 4:**
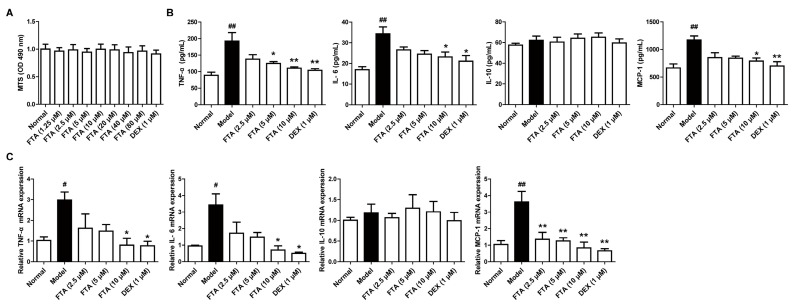
FTA reduces the expressions of cytokines in zymosan-stimulated RAW 264.7 cells. Cells were pretreated with zymosan (10 μg/mL) for 30 min, then treated with FTA (2.5, 5, 10 μM) and DEX (1 μM) for 4 h, and supernatants and total cells were collected. (**A**) Effect of FTA on the cell viability of macrophage RAW 264.7 cells. Cells were incubated with or without FTA (1.25–80 μM) and DEX (1 μM) for 24 h. Cell viability was determined by MTS; (**B**) The protein of cytokines were determined by ELISA; (**C**) Cytokine mRNA levels were quantified by QRT-PCR. Data were expressed as means ± SEM (*n* = 3). * *p* < 0.05, ** *p* < 0.01 vs. Model; ^#^ *p* < 0.05, ^##^ *p* < 0.01 vs. Normal.

Increased cytokine secretion may be responsible for the inflammatory process, including neutrophil migration [21], and macrophages are a significant source of cytokines [22]. To further explore the anti-inflammatory action and mechanisms of FTA, we researched the effect on the cytokines expressions in zymosan-stimulated macrophages. We firstly tested FTA cytotoxicity against macrophages. It was shown that FTA (1.25–80 μM) and DEX (1 μM) did not affect the viability of macrophage RAW 264.7 cells (Figure 4A). Results from the ELISA demonstrated that the zymosan stimulation led to a notable increase in protein levels of proinflammatory cytokines (TNF-α and IL-6) and chemokine (MCP-1), except for anti-inflammatory agent (IL-10). FTA (10 μM) and DEX (1 μM) displayed a striking reduction of the above cytokines (Figure 4B). Similar results were observed in mRNA expressions of cytokines in RAW 264.7 cells (Figure 4C).

### 2.4. FTA Inhibits Zymosan-Induced Inflammatory Mediators through Activating NF-κB

**Figure 5:**
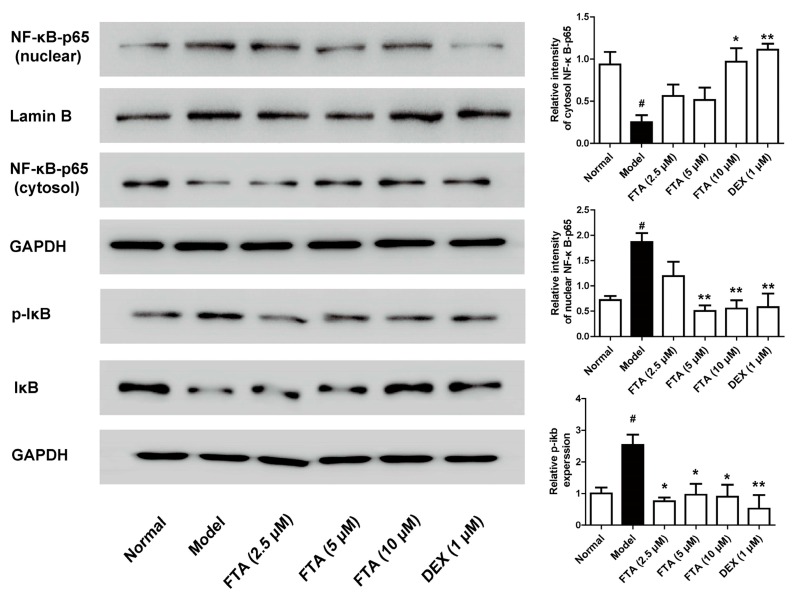
Effect of FTA on NF-κB signaling pathway activation in zymosan-stimulated macrophage RAW 264.7 cells. Cells were treated with FTA or DEX in the presence or absence of zymosan (10 μg/mL). NF-κB-p65 and IκB expression were detected by Western blotting. Data were expressed as means ± SEM (*n* = 3). * *p* < 0.05, ** *p* < 0.01 vs. Model; ^#^ *p* < 0.05 vs. Normal.

It is pointed out that NF-κB is essential for the transcriptional regulation of inflammatory mediators [23]. Therefore, we employed the influence of FTA on NF-kB signaling pathway. As Figure 5 showed, zymosan exposure significantly increased IκB and p65 NF-κB protein phosphorylation. FTA (10 μM) treatment remarkably inhibited zymosan-induced IκB and p65 NF-κB protein phosphorylation.

## 3. Discussion

FTA, a phenylethanoid glycoside isolated from *Forsythia suspensa*, has significant pharmacological activity, particularly anti-inflammatory [24], antitumor [25], anti-endotoxin [26], and antioxidant [27]. In this research, we ascertained the effect of FTA on zymosan-induced peritonitis in mice, a commonly used animal model of acute resolving inflammation. Zymosan injection induced neutrophil influx at 4 h of peritonitis, which was agree with the previous study [20]. As expected, we could observe a strong decline in neutrophil numbers in the peritoneal lavage after FTA treatment.

Zymosan induces the expressions of inflammatory cytokines, including TNF-α, IL-6, and IL-10, which are pivotal in inflammation [28]. After acute injury, TNF-α, characterized by the earliest and large releases in a short time, is a main endogenous mediator of host responses. IL-6, a pleiotropic cytokine produced by various cells such as T cells, macrophages and fibroblast, is involved in the regulation of the vascular inflammation and immune response [29]. IL-10 is an anti-inflammatory cytokine and suppresses inflammatory response by increasing anti-inflammatory factors and reducing proinflammatory factors, as well as inhibiting the activation and function of T cells, macrophages, and monocytes [30,31,32]. Moreover, such cytokines are associated with the neutrophil infiltration in zymosan-inflamed peritoneum [21]. Our results showed a decrease in quantity of neutrophil cells in peritoneal cavity in FTA treated mice, pointing out that the FTA-induced reduction in the number of peritoneal neutrophils may be relevant to inhibition of cell migration. The present study also proved that FTA reduced the levels of TNF-α and IL-6 in the peritoneal exudate in mice, but not IL-10 level, which was the same with other groups [21,33].

The CC chemokine MCP-1 is one of the most important potent chemotactic factor for leukocytes and its elevation is important in the progression of neutrophil migration to the site of tissue injury [34,35]. In the early migration, neutrophils are mainly associated with inflammatory signals induced by local macrophages [36]. FTA could reduce the production of MCP-1, which may result in neutrophil infiltration.

NF-κB is a critical part in regulating inflammatory mediator production and has an important function in zymosan-induced peritonitis [24]. During inflammation, NF-κB triggers the transcription of cytokines in their activated state [37]. FTA has been proved to restrain inflammatory cytokines in activated macrophages by inhibiting NF-κB signaling pathway [26]. Additionally, FTA has the neuroprotective effect in microglia cells by inhibiting NF-κB activation and IκBα degradation [38]. In vivo, FTA inhibited NF-κB activation in LPS/GalN-treated mice [19] and downregulated the NF-κB in the bursa of Fabricius of LPS-treated chickens [39]. It also dose-dependently inhibited CS-induced IκBα and p65 NF-κB phosphorylation in the lung tissues of CS-exposed mice [40]. The present findings allowed us to postulate FTA exhibited the anti-inflammatory action via the inactivation of NF-κB. To verify this hypothesis, we detected the effects of FTA on zymosan-induced the activation of NF-κB in RAW 264.7 cells. The experiments showed that FTA could antagonize NF-kB activation induced by zymosan. These suggested that FTA inhibited zymosan-induced acute inflammatory response by inhibiting the NF-κB signaling pathway.

## 4. Materials and Methods

### 4.1. Drugs and Chemicals

Forsythoside A (FTA, purity > 98%) was purchased from the National Institutes for Food and Drug Control (Beijing, China). Dexamethasone (DEX) was obtained from Zhejiang Xianju Pharmaceutical Co., Ltd. (Taizhou, China). Zymosan A from *Saccharomyces cerevisiae* was purchased from Sigma-Aldrich (St. Louis, MO, USA). Anti-mouse mAbs Gr-1-FITC and F4/80-PE were purchased from Biolegend (San Diego, CA, USA). The enzyme-linked immunosorbent assay (ELISA) kits for TNF-α, IL-6, IL-10, and MCP-1 were purchased from ExCell Biotech (Taicang) Co., Ltd. (Shanghai, China). Real-time PCR kits were purchased from Shanghai Hifun Biotechnology Co. Ltd. (Shanghai, China). Antibodies against NF-κB-p65, p-NF-κB-p65, IκBα, and p-IκBα were purchased from Cell Signaling Technology, Inc. (Boston, MA, USA).

### 4.2. Animals

The study was conducted on 6-week-old male mice of C57BL/6J purchased from Beijing Vital River Laboratory Animal Technology Co., Ltd. The animals were kept in standard conditions: temperature 20 ± 2 °C, 12:12 LD, with ad libitum access to the consumption of water and food. All mouse experiments followed protocols approved by the Institutional Animal Care and Use Committee in Institut Pasteur of Shanghai (No. SZY 20170913, Shanghai University of Traditional Chinese Medicine, Shanghai, China).

### 4.3. Zymosan-Induced Acute Peritonitis in Mice

To examine FTA anti-inflammatory action, mice were randomly divided into six groups: normal group, model group, FTA (10, 20, 40 mg/kg) groups, and DEX (4 mg/kg) group after one week of acclimatization. Animals received FTA (10, 20, 40 mg/kg) or DEX (4 mg/kg) i.p. injection 30 min after i.p. zymosan injection. Zymosan A was dissolved in sterile 0.9% *w*/*v* saline (2 mg/mL) before treatment and 0.5 mL was injected intraperitoneally. FTA (10, 20, 40 mg/kg) or DEX (4 mg/kg) was injected intraperitoneally 30 min after the administration of zymosan. Normal and model mice received an equal volume of vehicle according to the same schedule. Animals were sacrificed 4 h after zymosan A treatment. The peritoneal lavage fluid was managed for flow staining (2.5 million). The blood was centrifuged to get supernatant for ELISA. Cell pellets, fluids, and serum were stored at –80 °C until further analysis.

### 4.4. Antibodies and Flow Cytometry Analysis

For flow cytometric analysis of cell populations in the peritoneal cavity, cell suspensions (2.5 million) were centrifuged (2000 rpm, 5 min, 4 °C), washed and stained with anti-mouse mAbs Gr-1-FITC and F4/80-PE (all from Biolegend) for 30 min at room temperature. Data were acquired on BD fluorescence-activated cell sorting LSR-II (BD Biosciences) and analyzed with Flowjo software (TreeStar, Ashland, OR, USA).

### 4.5. Cytokines and Chemokine Detection 

To assess the level of TNF-α, IL-6, IL-10, and MCP-1 released in both peritoneal exudates and zymosan stimulated RAW 264.7 macrophages, a commercial ELISA kit was used (ExCell Biotech, Taicang).

### 4.6. Cell Culture and Treatment

RAW 264.7 cells were purchased from Shanghai Institutes for Biological Sciences (Shanghai, China). Cells were grown in DMEM supplemented with 10% fetal bovine serum, 100 units/mL penicillin, and 100 mg/mL streptomycin. Cell cultures were maintained at 37 °C in a humidified atmosphere containing 5% CO_2_. The cells (1 × 10^6^ cells/well) were seeded into six-well plates and pretreated with zymosan (10 μg/mL) for 30 min, then treated with FTA (2.5, 5, 10 μM) and DEX (1 μM) for 4 h. Supernatants and cells were collected for further analysis.

### 4.7. MTS Assays

RAW 264.7 cells were seeded into 96-well plates at a density of 1 × 10^4^ per well (100 µL) and treated with the following: vehicle control, FTA at 1.25 to 80 µM and DEX at 1 μM. Wells with serum-free medium were used as negative control. The cells were incubated for 24 h. Thereafter, 3 h before the end of incubation, 10 µL of the MTS solution was added into each well. The absorbance of each well was read directly at 490 nm with a microplate reader (VersaMAx, Molecular Devices, Sunnyvale, CA, USA).

### 4.8. Quantitative Real-Time (QRT) PCR Assay

DNA from collected cells was reversed transcribed into cDNA using EZBioscienceTM EZ-press cell to cDNA Kit. Real-time PCR assay was performed with a 7500 Real-Time PCR System using EZBioscienceTM qPCR SYBR Green Master Mix. The primers used were: TNF-α, forward: 5′-TCTTCTCATTCCTGCTTGTGG-3′, reverse: 5′-GGTCTGGGCCATAGAACTGA-3′; IL-6, forward: 5′-GGAGCCCACCAAGAACGATAG-3′, reverse: 5′-GTGAAGTAGGGAAGGCCGTG-3′; IL-10, forward: 5′-CAGAGCCACATGCTCCTAGA-3′, reverse: 5′-TGTCCAGCTGGTCCTTTGTT-3′; MCP-1, forward: 5′-GGCTCAGCCAGATGCAGTTAA-3′, reverse: 5′-CCTACTCATTGGGATCATCTTGCT-3′; and GAPDH, forward: 5′-GGTGAAGGTCGGTGTGAACG-3′, reverse: 5-CTCGCTCCTGGAAGATGGTG-3′.

### 4.9. Western Blotting

Nuclear and cytoplasmic protein were extracted using a nuclear and cytoplasmic protein extraction kit (Beyotime, Shanghai, China). The whole cell lysates were prepared by suspending cells in NP40 buffer (Beyotime, Shanghai, China). Protein extracts were quantified by bicinchoninic acid (BCA) protein assay. Samples were resolved by SDS-PAGE and further transferred to polyvinylidene fluoride (PVDF) membranes. After being blocked for 2 h at room temperature with 5% nonfat milk, the membranes were incubated with different antibodies overnight at 4 °C, and then rinsed and incubated with a secondary antibody for 1 h at 37 °C. Detection was using Tanon-4200SF (Tanon Science and Technology Co., Ltd., Shanghai, China).

### 4.10. Statistical Analysis

The values are expressed as means ± SEM. Statistical comparisons were made using one-way analysis of variance (ANOVA) and post hoc Tukey’s test. *p* values less than 0.05 were considered to be indicate a statistically significant difference.

## 5. Conclusions

In conclusion, we demonstrate that FTA inhibits zymosan-induced inflammatory responses through decreasing neutrophil numbers, and the production of TNF-α, IL-6, and MCP-1. This effect is achieved by inhibiting NF-κB activity. This evidence suggest that FTA has a potential pro-resolving activity for the treatment of acute inflammation. To further understand the therapeutic effect of FTA on zymosan-induced acute inflammation, additional experiments on the FTA-mediated prevention of zymosan-induced neutrophil infiltration are required in the future.
